# Challenges and strategies to research ethics in conducting COVID‐19 research

**DOI:** 10.1111/jebm.12388

**Published:** 2020-05-22

**Authors:** Xitao Ma, Yanqiao Wang, Tian Gao, Qing He, Yan He, Rensong Yue, Fengming You, Jianyuan Tang

**Affiliations:** ^1^ Hospital of Chengdu University of Traditional Chinese Medicine Chengdu P.R. China

**Keywords:** COVID‐19, ethical review, informed consent, public health emergency

## Abstract

The number of research involving human subjects on coronavirus disease 2019 (COVID‐19) is surging, bringing challenges to the ethical review committee (ERC) in terms of reviewing speed and special ethical considerations under the pandemic. However, the existing ethical review system and regulations have their limitations to meet the demand for a prompt and efficient epidemic control. Since the research under the public health emergency is different from that carried out in familiar situations to design and implementation, the strategy for a satisfactory ERC response should balance the duty of protecting individual participants as well as the special public needs derived from the disease control. It is suggested that the ethical review‐related regulations need to be updated, and a unified supervision system to the overall ERC is required. ERC collaboration, capacity‐improving and efficiency‐improving measures need to be taken. With respect to the reviewing guidelines, it is suggested that the international norms should be explained with more consideration of the local condition and the exceptional circumstances in this public health emergency. A joint effort needs to be taken for better research conduction.

## 1 INTRODUCTION

In December 2019, an epidemic with pneumonia as the major manifestation of the 2019 novel coronal virus infection first broke out in Wuhan, China, imposing massive impact on China and the world.^1^, ^2^ This new coronavirus is highly infective, threatening public health and safety. The disease was so severe that as early as 30 January 2020, the outbreak was declared by WHO as a Public Health Emergency of International Concern. It was unprecedented and our understandings to the virus and the disease are quite limited. Currently, there are no vaccines or antiviral treatments that are proved to be effective for the management of the COVID‐19. Various scientific studies have been conducted to form a concise picture about its occurrence, development, and prognosis and have finally found effective measures for prevention, diagnosis, and treatment.

Widely recognized by the international community, ethical review plays an important role in protecting the rights, safety, and well‐being of the research participants and ensuring the scientific correctness. In the current grim situation of the epidemic, the authors believe that in the COVID‐19 emergency condition, the exceptional circumstances of the pandemic should justify the use of unproven interventions just like the WHO panel considered in 2014 for the Ebola disease.^3^ Therefore, the ethical review committee (ERC) should explain the general ethical principles more flexibly, streamline the work, and facilitate its procedure as a response if it allows. The priority to the protection of life and the pursuit of effectiveness should always be kept in mind. Therefore, the research during an unusual period can be reviewed, and informed consent can be obtained in a timely and reasonable manner.

## 2 CHARACTERISTICS OF RESEARCHES ON COVID‐2019

Scientific research is of great significance to quickly understand the COVID‐19 when there is no definitive treatment for it, thus promoting the emergency response. On 30 January 2020, the WHO issued temporary recommendations that we should enhance public health measures for containment of the current outbreak, continue to identify the zoonotic source of the outbreak, and particularly the potential for circulation, and conduct investigations to understand the epidemiology and evolution of this outbreak and measures to contain it; the recommendations also noted that all countries should place particular emphasis on contributing to the international response through multisectoral communication and collaboration and active participation in increasing the knowledge on the virus and the disease, as well as advancing research and that the global community should support the research for developing necessary treatment. Different from the researches under the common circumstances, studies conducted during the epidemic have their own characteristics^4^: most of the researches are based on the clinical treatment, and the objective is to bring relief to attack by the epidemic, as opposed to generating results of universality. Furthermore, the critical shortage of resources and researchers,^5^, ^6^ especially the front‐line medical staff and public health professionals shouldering responsibilities of both treatment and research, hinders the feasibility of researches.

As of 10 February 2020, the authors found 69 registered researches concerning the COVID‐19 on the website of the Chinese Clinical Trial Registry (http://www.chictr.org.cn). These include interventional, diagnosis, and observational studies. As for the design of clinical trials, many are epidemiological investigations, cross‐sectional studies, cohort studies, single‐arm studies, and real‐world studies, in addition to the small amount of double‐blind randomized clinical trials, which can be partly attributed to the shortage of resources, treatment pressure, and the treatment objective for the majority of trials, minimizing the possibility to conduct such rigorous trials.

A total of 96 articles on COVID‐19 were retrieved from PubMed on 12 February 2020, the majority of which are descriptive researches using epidemiological methods, depicting variation of symptoms and signs of different regions,^7^, ^8^ various stages of the disease, different groups, and some predicting the trend of disease.^9^ Studies on onvirology,^10^ gene,^11^ and diagnosis also occupy considerable proportions. Researches on the treatment are at large the descriptive studies, such as clinical observations and case reports. The variance in the number of these research types reflects the efforts devoted to the containment of the disease.

## 3 ETHICAL REVIEW OF RESEARCH UNDER COVID‐19 EPIDEMIC

It's widely known that an ethical review is required before the launch of any biomedical research involving human subjects. The ethical review for researches of COVID‐19 at this time is at “exceptional circumstances” for a series of special considerations, such as the magnitude of the epidemic, its contagiousness, additional burdens on health systems, and the limited time left to the investigators just like the consensus that the WHO ethics panel of 2014 reached during the Ebola epidemic^3^ that clinical trials had to move forward without undue delays. When infectious diseases break out, the design and conduction of research are different from usual ones.^4^ The ERC members are expected to fully comprehend the concrete implications of the international guidelines for ethical review, to provide pragmatic ethical support to these studies, with specific consideration of the local condition of the subjects and the researchers under the public health emergency.

In China, the basis for ethical review mainly resides in the *Guidelines for Ethical Review Work of Drug Clinical Trials* issued by the China Food and Drug Administration in 2010 and *Measures for the Ethical Review of Biomedical Research Involving Humans* issued by the Ministry of Health in 2016, which, however, contain no regulations related to the ethical review of researches involving humans under the public health emergency. To conduct the review routinely will fail to meet the promptness requirement in an epidemic, while to conduct the expedited review may be criticized for procedural issues.^3^ Researchers and reviewers can also be referred to the *Helsinki Declaration* (2013) by the World Medical Association and the *International Ethical Guidelines for Biomedical Research Involving Human Subjects* (2016) by the Council for International Organizations of Medical Sciences (CIOMS), *Guidance for managing ethical issues in infectious disease outbreaks* (2016) by the WHO. Nevertheless, the review quality varies because of the discrepant understanding of the terms due to different backgrounds and expertise of committee members, leading to an impact on the consistency of the review results. A specific case of such a dilemma is analyzed below.

### 3.1 Case

Is informed consent necessary for epidemiological investigation of COVID‐19 using electronic medical record?

### 3.2 Applicable Norms

#### 3.2.1 Paragraph 32 in *Declaration of Helsinki* (2013)

For medical research using identifiable human material or data, such as research on material or data contained in biobanks or similar repositories, physicians must seek informed consent for its collection, storage, and/or reuse. There may be exceptional situations where consent would be impossible or impracticable to obtain for such research. In such circumstances, the research may be done only after consideration and approval of a research ethics committee.

#### 3.2.2 Guidelines from CIOMS

Research in disasters and disease outbreaks in *International Ethical Guidelines for Health‐related Research Involving Humans* from CIOMS: ethical principles embodied in these Guidelines. Researchers, sponsors, international organizations, research ethics committees, and other relevant stakeholders should ensure that the individual informed consent of participants is obtained even in a situation of duress, unless the conditions for a waiver of informed consent are met.

### 3.3 Claim

The investigation uses the medical history, and the identifiable human data are involved, so the ethical review is mandatory. Informed consent obtaining is not impossible in the epidemic. CIOMS also claims that an ethics committee should not approve a waiver of informed consent because of obtaining difficulties. To get the informed consent and signature, if possible, it is required to ensure that the interests of these subjects are being served.

### 3.4 The counter claim

All researches in the epidemic are racing against time. Risks involved in investigations with electronic history are minimal; the arrangement of informed consent and the signature seems to be burdensome; medical personnel as the researchers will be more likely to be infected, entailing more risks than benefits.

### 3.5 Reflection: ethical review in a public health emergency should take special circumstances into account

Ethical review is an institutional tool recognized for the safe conduction of scientific research in modern society; yet, the institution and the regulation were formulated in a special historical and social background and were based on the routine research administration and the individualism. During an epidemic, the ethical committees are obliged to seek the substantial results and give priority to life, and to balance the relationship between individual and the collective when conducting a review, taking the special circumstances into account together with the rights and interests of the subjects. Every minute counts during an epidemic; research in such background is for the benefit of both the individual subject and the public in the affected area. An investigation based on the data from the electronic medical record poses no direct medical threats to the subjects. Adherence to the principles of privacy protection in the research can well balance the research risks and benefits, minimize the burden on researchers, so as to conduct the research promptly and effectively, thus saving the life of more people.

### 3.6 Suggestion

With the above‐mentioned principles of seeking substantial results and giving priority to life, the ethical committee can decide to streamline the review algorithm by removing temporarily the informed consent procedure or just the signature of the consent. In the specific conduction of an investigation, oral informed consent can be employed first, and the written consent can be signed after the subject is recovered from the disease and released from quarantine, if possible. In addition, if one changes his or her mind and refuses to sign the informed consent, then his or her data can be excluded from the research. Hence, the informed consent right of the potential subjects is respected. Signature obtained afterward can also minimize the infection risk during research, protecting the life security of the researchers and the public.

## 4 REFLECTIONS ON THE ETHICAL REVIEW IN THE BACKGROUND OF COVID‐19

The ethical review for researches on COVID‐19 involving biological specimens or data should abide by relevant laws and regulations in China. However, the demand for a timely ERC respond contradicts with that of the current ethical regulations in China with regard to procedure specifically. Moreover, the ERC in China is lacking a unified supervision system and the ERC capacity is not evaluated in due time, which may cause undue delay of a potential scientific protocol.

### 4.1 The gap in existing laws and regulations for scientific research management in the context of major public health emergencies needs to be filled

On 20 January 2020, the government of China classified COVID‐19 epidemic into the class B infectious diseases and decided to control it as a class A infectious disease; thus, the emergency responses from all government agencies and organizations have concrete laws to abide by. But the *Law of the People's Republic of China on Prevention and Treatment of Infectious Diseases (2013 Amendment)* does not discriminate the clinical treatment and the scientific research in the very period of an epidemic. Article 39 of the law says when encountered with a patient with class B or C infectious disease, the medical and health institutions shall treat the patient based on his or her condition and take action to control the spread of the disease. Besides, article 51 of the law goes like this: the medical and health institutions shall take measures to improve the capacity of cure by the diagnosis standards and the treatment requirement of the public health authority under the state council. Therefore, treatment options to patients in an epidemic largely depend on the decision of the medical and health institutions. Before the vaccine is available, the recommendations to treatment are based on the pre‐existing knowledge and are “investigative” to a certain extent. For the infected, different from the informed consent choice under normal conditions, to accept the treatment from institutions is exactly the performance of his or her obligation to the law under emergency condition. So for research conducted in disease outbreak which is to evaluate the efficacy and safety of a certain investigation, is ethical review oversight necessary? If it's a must, the ethical review for the public health emergency should be different from that under common situation. Health administrations should support the ERCs in their attempts to develop expedited procedures adaptive to the specific need of health emergencies in the form of regulations or policies.

### 4.2 Special considerations to research ethics in the epidemic

In the epidemic, front‐line medical personnel are shouldering responsibilities for both everyday diagnosis and treatment and scientific research. It is difficult for them to balance the life‐saving obligation with scientific observation, informed notification, and records keeping.^6^ Thus, ERC is suggested to assess the risk/benefit ratio and the way of informed consent in this occasion by comparing the individual interest and collective interest besides general ethical considerations.^12^

Different kinds of researches may be conducted during the epidemic, including randomized controlled trials (RCTs) bearing the highest evidence‐based level, prospective observational studies, cross‐sectional studies, real‐world studies, and retrospective studies using human specimen or data from electronic medical records. Therefore, the risks are different. The risk of RCT is generally high, and therefore informed consent should be mandatory even on this occasion. Since the demand for a qualified RCT is extremely high, the RCT design should not be encouraged in this limited resource condition, unless it is of great importance and is in real need just like the scientists called for recently. Therefore, ERC has to evaluate the local situation of the research site to human resources, sample size, and so on, besides the general ethical considerations.^13^ However, other observational studies can be considered to review with an expedited procedure with informed consent and/or signature waived. This can be justified from the ethical aspect with comparisons of individual needs and collective interest.

### 4.3 The ethical review regulation needs to be updated, and ERC cooperation should be strengthened

According to the current guidelines in China, meeting review is the major reviewing method for researches with more than minimal risks and a valid meeting review requires approval from more than half of the present committee members. In the face of COVID‐19 research, teleconference and videoconference review have already been widely applied by ERC; yet, many issues, such as sign‐in form, validity of electronic signature, and archiving of the ERC, need to be recognized by the regulations.

It's criticized that the lengthy ethical review procedures and communication between ERCs were important reasons for the delay in the commencement of research in the Ebola virus disease outbreak stage.^12^ Hence, the importance of efficient ERC collaboration and the quick response from the local ERCs are much emphasized to mitigate the disease harm especially in front of public health emergencies.^14^, ^15^, ^16^ Models for coordination and communication between ERCs and templates for expediting review of research protocols in epidemic and emergency conditions are being developed,^17^ providing useful information for the followers to learn. Additionally, the quality or capacity of the local ERC is crucial for the initiation of a pragmatic clinical trial which may adopt innovative and nontraditional methods other than traditional RCT design that goes beyond the ability of most ERC and may delay the research opportunities.^18^

In China, no consensus has been reached on the ethical cooperation of multi‐center clinical trials and there's no unified system to supervise the quality of single ERC. Therefore, China is in great need for an ethical review emergency response mechanism especially with regard to ERC collaboration in multisite clinical trials to contain the current and future possible public health emergencies.^18^, ^19^

## 5 CONCLUSION

The ethical review system has been introduced to China for over 20 years. However, there is no unified supervision agency or regulation for all the ERC. In the face of COVID‐19, the defects of lacking a systematic ethical reviewing system are demonstrated by the booming number of clinical trials. It is suggested that joint efforts should be taken for a sound and responsive, ethical reviewing system in front of the epidemic, namely, the construction of an effective ERC supervision system,^19^ the update of relevant regulations,^20^ and the continuous capacity improvement of the ERC.^21^
